# Induction of Apoptosis by Coptisine in Hep3B Hepatocellular Carcinoma Cells through Activation of the ROS-Mediated JNK Signaling Pathway

**DOI:** 10.3390/ijms21155502

**Published:** 2020-07-31

**Authors:** So Young Kim, Hyun Hwangbo, Hyesook Lee, Cheol Park, Gi-Young Kim, Sung-Kwon Moon, Seok Joong Yun, Wun-Jae Kim, Jaehun Cheong, Yung Hyun Choi

**Affiliations:** 1 Anti-Aging Research Center, Dong-Eui University, Busan 47340, Korea; 14731@deu.ac.kr (S.Y.K.); hbhyun2002@pusan.ac.kr (H.H.); 14769@deu.ac.kr (H.L.); 2 Department of Molecular Biology, Pusan National University, Busan 46241, Korea; 3 Department of Biochemistry, Dong-Eui University College of Korean Medicine, Busan 47227, Korea; 4 Division of Basic Sciences, College of Liberal Studies, Dong-Eui University, Busan 47340, Korea; parkch@deu.ac.kr; 5 Department of Marine Life Sciences, School of Marine Biomedical Sciences, Jeju National University, Jeju 63243, Korea; immunkim@jejunu.ac.kr; 6 Department of Food and Nutrition, Chung-Ang University, Anseong 17546, Korea; sumoon66@cau.ac.kr; 7 Department of Urology, College of Medicine, Chungbuk National University, Cheongju 28644, Korea; sjyun@chungbuk.ac.kr (S.J.Y.); wjkim@chungbuk.ac.kr (W.-J.K.)

**Keywords:** apoptosis, coptisine, DNA damage, Hep3B cells, c-Jun N-terminal kinase, reactive oxygen species

## Abstract

Hepatocellular carcinoma (HCC) has a high mortality rate worldwide, and treatment is very limited due to its high recurrence and low diagnosis rate, and therefore there is an increasing need to develop more effective drugs to treat HCC. Coptisine is one of the isoquinoline alkaloids, and it has various pharmacological effects. However, the evidence for the molecular mechanism of the anticancer efficacy is still insufficient. Therefore, this study investigated the antiproliferative effect of coptisine on human HCC Hep3B cells and identified the action mechanism. Our results showed that coptisine markedly increased DNA damage and apoptotic cell death, which was associated with induction of death receptor proteins. Coptisine also significantly upregulated expression of proapoptotic Bax protein, downregulated expression of anti-apoptotic Bcl-2 protein, and activated caspase-3, -8, and -9. In addition, coptisine remarkably increased the generation of reactive oxygen species (ROS), loss of mitochondrial membrane potential (MMP), and release of cytochrome *c* into the cytoplasm. However, *N*-acetylcysteine (NAC), a ROS scavenger, significantly attenuated the apoptosis-inducing effect of coptisine. It is worth noting that coptisine significantly upregulated phosphorylation of ROS-dependent c-Jun N-terminal kinase (JNK), whereas treatment with JNK inhibitor could suppress an apoptosis-related series event. Taken together, our results suggest that coptisine has an anticancer effect in Hep3B cells through ROS-mediated activation of the JNK signaling pathway.

## 1. Introduction

Hepatocellular carcinoma (HCC) is a major form of liver malignancy, which is the second highest cause of cancer death worldwide [1,2]. In the progress of HCC, cirrhosis and chronic liver disease are the most critical risk factors including hepatitis B and C virus infection, aflatoxin B1 intoxication, excessive alcoholism, and metabolic diseases [3,4]. Paradoxically, although the risk factors of HCC are quite well known and there are new and advanced technologies of diagnosis and treatment, the incidence and mortality of HCC continue to rise [5]. Early diagnosis is the key to obtaining the best outcomes for the cure of HCC, but treatment often fails, because at the beginning the symptoms are elusive [1]. For this reason, the concept of “early cancer”, which has been successfully applied to other malignancies, such as gastric cancer, has not yet been proven for HCC [5]. In addition, there are several treatment options used in the cure of HCC, such as surgical ablation and chemotherapy, but these are limited due to their side effects, complexities, and resistance to the available chemotherapy [6,7]. Therefore, at present, the most effective strategy for reducing mortality due to HCC is prevention, and it is considered that alternative therapies are needed to improve survival and quality of life [8,9].

Over recent decades, natural plant products, including medicinal herbs and phytochemicals, have been reported to have many advantages, due to their cost effectiveness, few adverse effects, and higher safety, and they have been used as potent therapeutic and chemopreventive agents for various chronic diseases [10,11]. Accumulated evidence suggests that several natural plant products can inhibit the progression of liver cancer through eliminating the known leading risk factors for HCC [7,10,11]. Coptisine is a type of isoquinoline alkaloid extracted from *Rhizoma coptidis* (roots of *Coptis chinensis* Franch) [12,13], and its pharmacological properties against inflammation [14], bacterial infection [15], and metabolic diseases [16,17] are well established. Recent studies have shown that this compound has an anticancer effect on lung, colorectal, breast, and liver cancers [18,19,20,21,22]. For example, Han et al. [19] demonstrated that coptisine induced apoptotic cell death through reactive oxygen species (ROS) production, a decline of mitochondrial membrane potential (MMP, ΔΨm), and activation of mitochondrial-associated apoptosis in human colon cancer. More current reports have suggested that coptisine inhibited cell growth, migration, and invasion via regulation of the phosphoinositide 3-kinases (PI3K)/Akt pathway in colorectal and pancreatic cancers [23,24]. Although several studies have shown that coptisine had anticancer activity against HCC, the underlying mechanism was unclear. Therefore, the purpose of this study was to investigate the anticancer effect of coptisine on human HCC Hep3B cells and to confirm the mechanism of action.

## 2. Results

### 2.1. Coptisine Inhibits Cell Viability and Induces Apoptosis in Hep3B Cells

**Figure 1:**
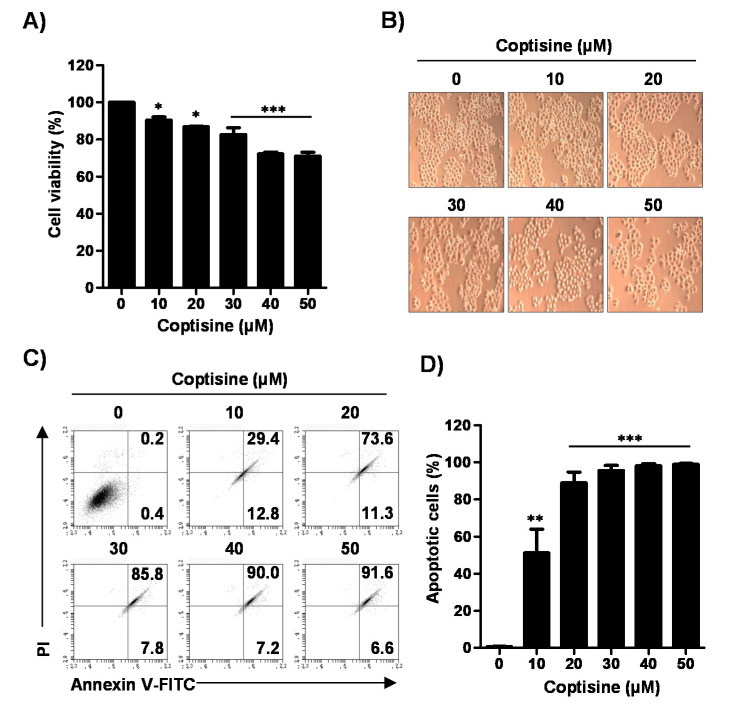
Effects of coptisine on cell viability and apoptosis in Hep3B cells. Hep3B cells were treated with the indicated concentrations of coptisine for 24 h. (**A**) After treated with coptisine, added the a 3-(4,5-dimethylthiazol-2-yl)-2-,5-diphenyltetrazolium bromide (MTT) solution, reacted for 2 h and then detected cell viability; (**B**) The morphology of Hep3B cells by coptisine were observed by phase-contrast microscopy (magnification, ×50); (**C**) The cells were harvested and stained with annexin V-fluorescein isothiocyanate (FITC) and propidium iodide (PI). After staining, cells were analyzed the apoptosis by flow cytometry. Annectin V-FITC results represent early apoptosis (lower right quadrant), and late apoptosis (upper right quadrant); (**D**) Statistical analysis of annexin V positive cells (** *p* < 0.01, and *** *p* < 0.001 vs. control). The results are presented as the mean ± standard deviation (SD) of three independent experiments.

To assess the cytotoxic effect of coptisine in Hep3B cells, we measured cell viability using a 3-(4,5-dimethylthiazol-2-yl)-2-,5-diphenyltetrazolium bromide (MTT) assay. As shown in Figure 1A,B, coptisine significantly inhibited the cell viability in a dose-dependent manner. On the basis of these results, we investigated whether coptisine-mediated inhibition of cell viability was involved in apoptosis. Our results showed that coptisine markedly increased the annexin V-positive cells in a dose-dependent manner (Figure 1C,D), indicating that coptisine-induced growth inhibition was associated with the induction of apoptosis.

### 2.2. Coptisine Enhances DNA Damage in Hep3B Cells

**Figure 2:**
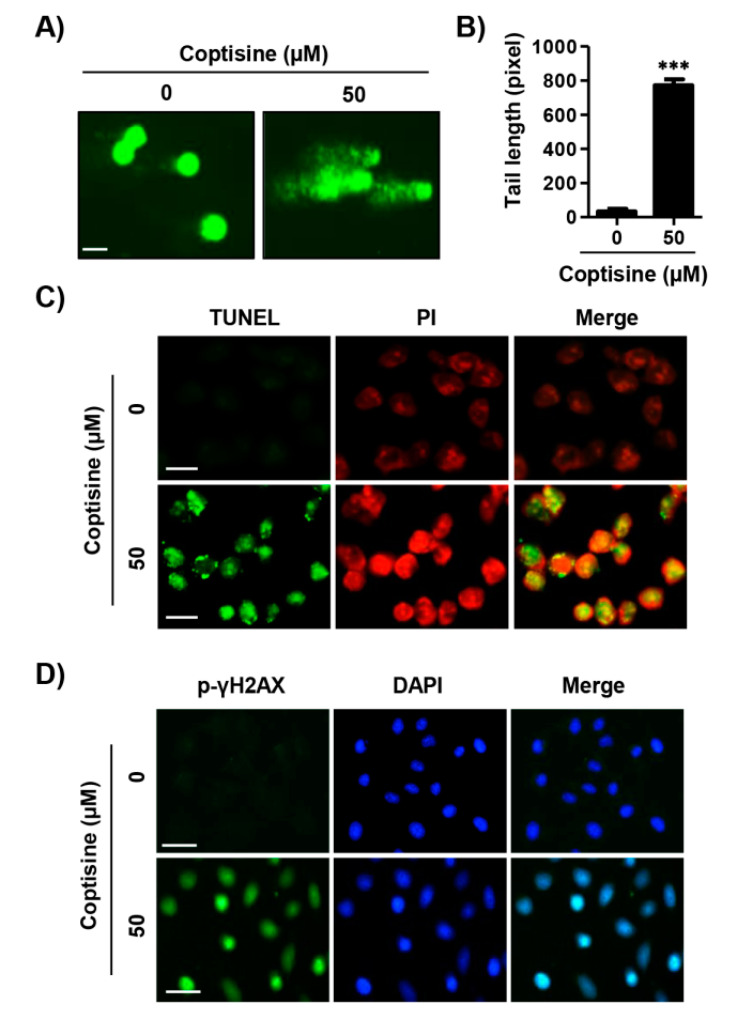
Induction of DNA damage by coptisine in Hep3B cells. Hep3B cells were treated with 50 µM coptisine for 24 h. (**A**) The comet assay was performed, and representative images of comet assay were taken by fluorescence microscopy. Scale bars, 50 µm; (**B**) Statistical analysis of tail length. *** *p* < 0.001 vs. control; (**C**) DNA break of apoptotic cells was assessed by terminal deoxynucleotidyl transferase-mediated dUTP-biotin nick end labeling (TUNEL) assay and images were obtained by fluorescence microscopy. Green staining (left) indicates apoptotic cells, and propidium iodide (PI) staining was used to visualize nuclei (middle). Scale bars, 20 µm; (**D**) The expression of phospho-H2A histone family member X (p-γH2AX) was investigated with immunofluorescence. Green staining (left) indicates p-γH2AX-positive cells and 4′,6-diamidino-2-phenylindole (DAPI) staining was used to visualize nuclei (middle). Merged image indicates the localization of p-γH2AX-positive cells in nuclei (right). Scale bars, 30 µm.

In order to evaluate the effect of coptisine on apoptotic DNA damage, comet assay, terminal deoxynucleotidyl transferase-mediated dUTP-biotin nick end labeling (TUNEL) assay, and immunofluorescence analysis of phospho-H2A histone family member X (p-γH2AX) were performed. Figure 2A shows that the coptisine-treated cells demonstrated a smeared pattern of nuclear DNA with a comet tail, but control cells did not. As shown in Figure 2B, the tail length was increased by about 15.89 times as compared with the control group. In addition, we found that treatment of coptisine increased the population of TUNEL positive cells (Figure 2C) and phosphorylation of γH2AX, a marker for DNA damage was upregulated (Figure 2D).

### 2.3. Coptisine Activates both Extrinsic and Intrinsic Apoptosis Pathways in Hep3b Cells

**Figure 3:**
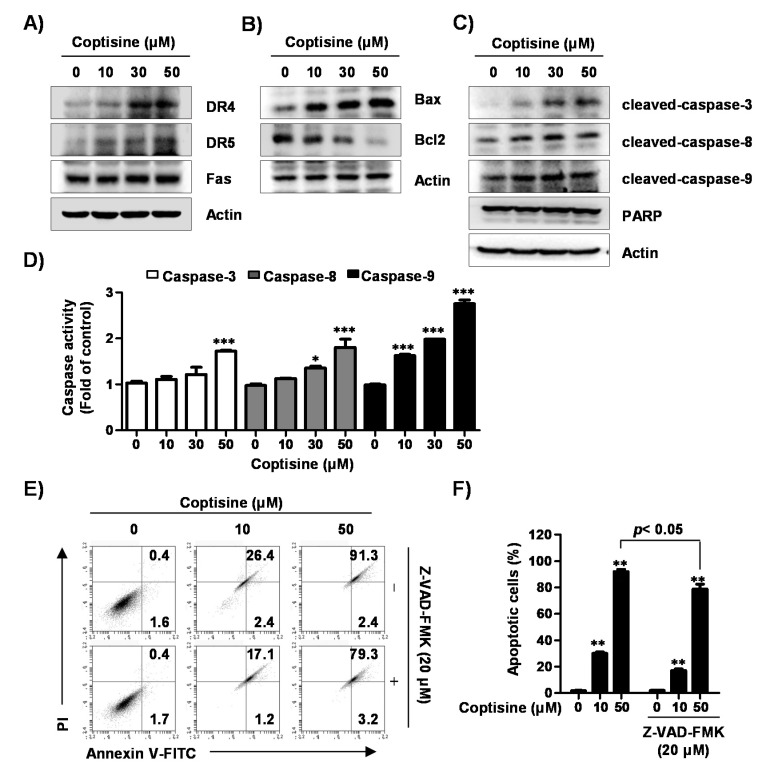
Effects of coptisine on extrinsic and intrinsic apoptotic pathways in Hep3B cells. (**A**–**C**) Hep3B cells were treated with coptisine for 24 h, cells were harvested for obtain total cell lysates. Cell lysates was separated by sodium dodecyl sulfate polyacrylamide gel (SDS-PAGE) and transferred to polymerization of vinylidene difluoride (PVDF) membranes. The membranes were blocked, probed with indicated antibodies, and incubated with secondary antibodies for detection; (**D**) After cells were treated with the indicated concentration of coptisine for 24 h, cells were harvested, and incubated with each substrate. The activities of caspases were detected by a microplate reader. Data are presented as the mean ± SD of three independent experiments. * *p* < 0.05 and *** *p* < 0.001 vs. control; (**E**) After cells were treated with benzyloxycarbonyl-Val-Ala-Asp (OMe) fluoromethylketone (Z-VAD-FMK), a pan caspase inhibitor, for 1 h, cells were treated with coptisine for 24 h. The cells were collected, stained with annexin V-FITC and PI, and analysis by flow cytometry; (**F**) Shows the percentage of annexin V+ cells. The results are presented as the mean ± standard deviation (SD) of three independent experiments. ** *p* < 0.01 vs. control.

Next, we investigated whether either extrinsic or intrinsic pathways were involved in coptisine-mediated apoptotic cell death. Our results demonstrated that coptisine precisely increased the expression of death receptor 4 (DR4) and DR5, but the expression of Fas remained unchanged (Figure 3A). Coptisine also certainly induced upregulation of proapoptotic protein such as Bcl-2-accociated X protein (Bax), as well as downregulation of anti-apoptotic Bcl-2 expression (Figure 3B). Furthermore, the expression of cleaved forms of caspase-3, caspase-8, caspase-9, and poly-ADP ribose polymerase (PARP) were markedly increased by coptisine treatment in a dose-dependent manner (Figure 3C), which was associated with increased activity of these caspases (Figure 3D). To confirm the effect of coptisine on caspase-dependent apoptosis, we used the benzyloxycarbonyl-Val-Ala-Asp (OMe) fluoromethylketone (Z-VAD-FMK), a pan caspase inhibitor and found that pretreatment of Z-VAD-FMK partially recovered coptisine-induced apoptosis (Figure 3E,F).

### 2.4. Coptisine Induces Mitochondrial Dysfunction in Hep3B Cells

**Figure 4:**
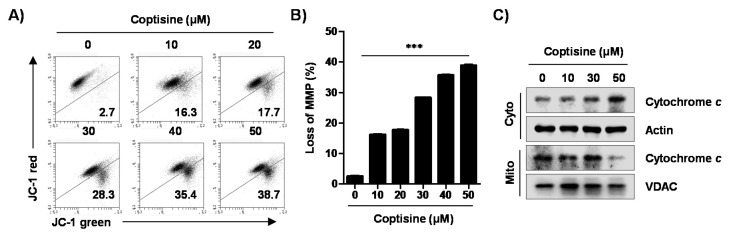
Induction of mitochondrial dysfunction and cytosolic release of cytochrome *c* by coptisine in Hep3B cells. (**A**,**B**) After treating the cells with the indicated concentration of coptisine for 24 h, the collected cells incubated with 10 µM 5,5′6,6′-tetrachloro-1,1′,3,3′-tetraethyl-imidacarbocyanine iodide (JC-1), and the values of mitochondrial membrane potential (MMP, ΔΨm) were evaluated using a flow cytometer. (**A**) depicts representative profile with percentage of JC-1 monomer (JC-1 green, lower right quadrant). (**B**) shows the percentage of MMP loss expressed as JC-1 green-positive cells. The data are expressed as the mean ± SD of three findings with independent experiments. *** *p* < 0.001 vs. control. In (**C**), after treatment with coptisine for 24 h, cytosolic and mitochondrial proteins were isolated, and analyzed for the expression of cytochrome *c* by Western blot analysis. Actin and voltage-dependent anion channel (VDAC) were used as loading controls for cytosol and mitochondrial fraction, respectively.

On the basis of the above result that coptisine regulated the expression of Bcl-2 family proteins, we investigated the effect of coptisine on the function of mitochondria in Hep3B cells. Consequently, the population of depolarized cell was substantially enhanced by coptisine (Figure 4A,B). Additionally, coptisine induced the release of cytochrome c from mitochondria to cytoplasm in a dose-dependent manner (Figure 4C).

### 2.5. Coptisine Increases Intracellular and Mitochondrial ROS Production in Hep3b Cells

**Figure 5:**
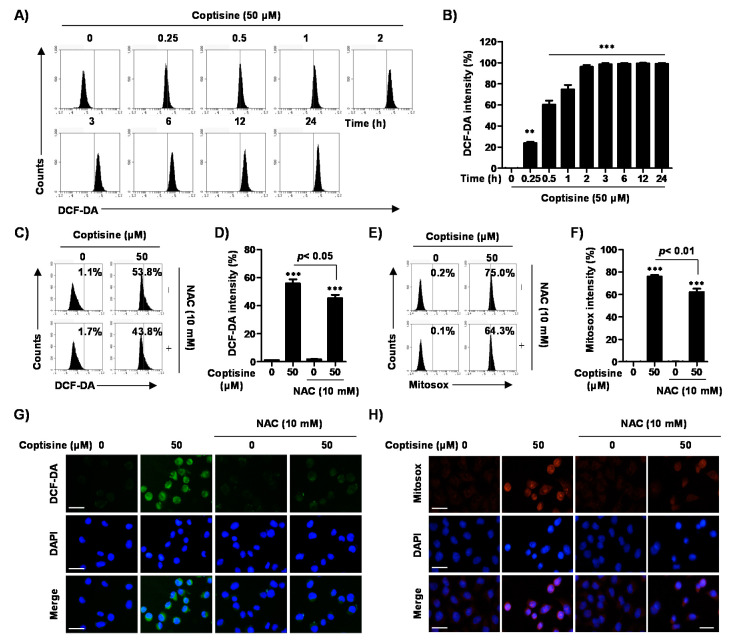
Increase of intracellular and mitochondrial reactive oxygen species (ROS) generation by coptisine in Hep3B cells. (**A**,**B**) The cells were treated with 50 µM coptisine for the indicated times. After staining with 10 µM 2′,7′-dichlorofluorescein diacetate (DCF-DA) for 20 min, the fluorescent intensity was analyzed by flow cytometry. (**A**) depicts representative profiles. (**B**) shows the fluorescent intensity, indicated as the percentage of all the present cells. The data are expressed as the mean ± SD of three findings with independent experiments. ** *p* < 0.01 and *** *p* < 0.001 vs. control. (**C**–**H**) Cells were pretreated with or without 10 mM *N*-acetylcysteine (NAC), a ROS scavenger, for 1 h, followed by coptisine treatment for 30 min. After being probed with 10 µM DCF-DA (**C**,**D**) or 1 µM MitoSOX™ red for 20 min (**E**,**F**), the stained cells were observed by flow cytometry (**C**–**F**) and fluorescent microscopy (**G**,**H**). (**C**,**E**) depict representative results. In (**D**,**F**), each bar indicates fluorescent intensity for DCF-DA and MitoSOX™ red. In (**G**,**H**), representative fluorescent images. DAPI staining was used to visualize nuclei. Scale bars, 30 µm.

To evaluate the coptisine-induced apoptosis due to the oxidative stress in Hep3B cells, we measured ROS generation by flow cytometric analysis. Figure 5A,B shows that coptisine significantly increased ROS generation from 15 min (22.53%) after treatment, and then maintained ROS levels for 24 h (97.84%). Therefore, the source of ROS generation by coptisine was reconfirmed, and we evaluated if the cytotoxic effect of coptisine was dependent on ROS. Our results showed that coptisine elevated the levels of intracellular ROS as well as mitochondrial ROS, which were identified by MitoSOX™ red staining, but the increase was markedly suppressed by *N*-acetylcysteine (NAC), a ROS scavenger (Figure 5C–F). In addition, the results of flow cytometry were consistent with that of fluorescence microscopy observation (Figure 5G,H).

### 2.6. Coptisine Regulates ROS-Dependent Apoptosis in Hep3b Cells

**Figure 6:**
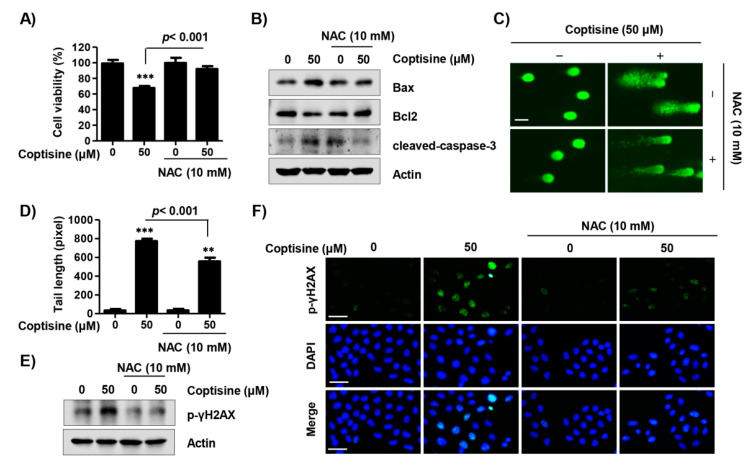
Induction of apoptotic cell death via ROS production by coptisine in Hep3B cells. Cells were pretreated with or without 10 mM NAC for 1 h followed by coptisine treatment for 24 h. (**A**) Cell viability was measured by an MTT assay. *** *p* < 0.001 vs. control; (**B**) The apoptosis regulatory proteins were detected by Western blot analysis; (**C**) Comet assay was performed, and representative images were taken by a fluorescence microscope. Scale bars. 50 µm; (**D**) The statistical analysis of tail length. ** *p* < 0.01 and *** *p* < 0.001 vs. control; (**E**,**F**) p-γH2AX expression was assessed by Western blot analysis and fluorescence microscopy. DAPI staining was used to visualize nuclei. Scale bars, 30 µm.

On the basis of the result that coptisine induced ROS production, we assessed whether coptisine-mediated apoptosis was either dependent on, or independent of ROS in Hep3B cells. Our findings showed that pretreatment of NAC significantly restored the reduced survival rate by coptisine to the control level (Figure 6A). In addition, upregulation of the expression of Bax and cleaved-caspase-3 in coptisine-treated cells were suppressed by NAC (Figure 6B). Moreover, NAC substantially inhibited the comet tail length (Figure 6C,D) and overexpression of p-γH2AX by coptisine (Figure 6E,F).

### 2.7. Coptisine Induces Apoptosis via Ros-Mediated Jnk Signaling Pathway in Hep3b Cells

**Figure 7:**
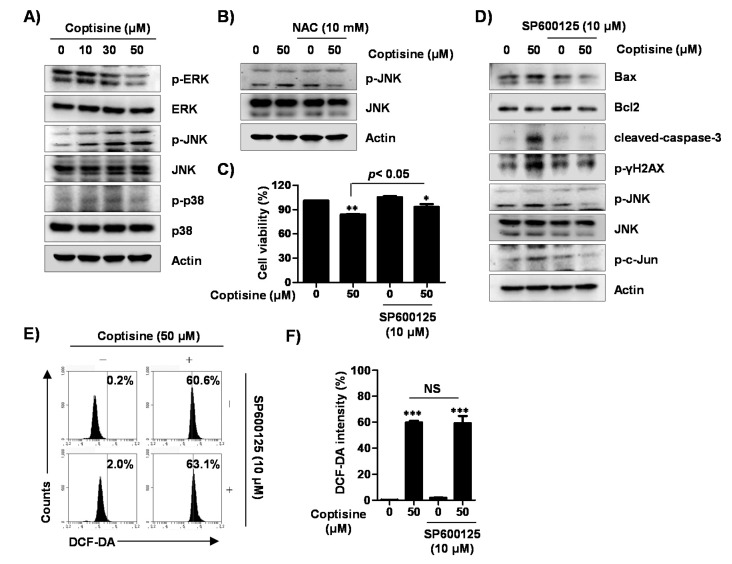
Role of c-Jun N-terminal kinase (JNK) signaling pathway in coptisine-induced apoptotic cell death in Hep3B cells. (**A**) Cells were treated with the indicated concentration of coptisine for 24 h, and then harvested. The isolated proteins were probed with primary specific mitogen-activated protein kinase (MAPK) antibodies; (**B**) Cells were pretreated with or without 10 mM NAC, for 1 h, followed by coptisine treatment for 24 h. Lysate proteins were subjected to anti-p-JNK and anti-JNK antibodies; (**C**,**D**) Cells were pretreated with or without 10 µM SP600125, for 1 h, before coptisine treatment for 24 h. In (C), cell viability was determined by MTT assay. The results are presented as the mean ± SD of three independent experiments. * *p* < 0.05 and ** *p* < 0.01 vs. control. In (D), expression of apoptosis-related proteins was analyzed by Western blot analysis; (**E**,**F**) The cells were pretreated with or without 10 µM SP600125 for 1 h before coptisine treatment for 30 min. Cells were harvested and stained with DCF-DA, and the ROS levels detected by flow cytometry. In (**E**), representative results. In (**F**), each bar indicates the fluorescent intensity for DCF-DA. The results are presented as the mean ± SD of three independent experiments. *** *p* < 0.001 vs. control. NS means not significant.

To identify the action mechanism of coptisine-induced apoptosis in Hep3B cells, we investigated the phosphorylation status of the mitogen-activated protein kinases (MAPKs) signaling pathway including the extracellular signal-regulated kinase (ERK), c-Jun N-terminal kinase (JNK), and p38 MAPK. The results demonstrated that coptisine upregulated the phosphorylation of JNK (c-JUN) with increasing concentration, while phosphorylated ERK (p-ERK) was decreased without affecting p38 MAPK (Figure 7A). Additionally, pretreatment of NAC gradually inhibited the overexpression of p-JNK by coptisine (Figure 7B). Furthermore, SP600125, a JNK inhibitor, slightly relieved the decrease in cell viability caused by coptisine (Figure 7C). SP600125 also reversed both the upregulation of expression of proapoptosis-related factors, and downregulation of anti-apoptotic Bcl-2 expression, following treatment by coptisine (Figure 7D). However, SP600125 did not change the coptisine-induced intracellular ROS levels (Figure 7E,F), suggesting that ROS can act upstream of JNK activation in coptisine-stimulated Hep3B cells.

## 3. Discussion

Coptisine is one of the active compounds discovered in *Rhizoma coptidis* [14]. Although its anticancer function has been reported for various types of cancers including HCC liver [18,19,20], the underlying mechanism for the anticancer activity of coptisine in HCC is unclear. Therefore, in the present study, we examined the anticancer effect of coptisine on HCC Hep3B cells and sought to identify the action mechanism. 

Apoptosis is a very tightly programmed cell death process that plays a crucial role in several biological processes in normal tissues. Defects in apoptosis can promote tumor development, and also make cancer cells resistant to treatment. In this respect, the evasion of apoptosis is a noticeable hallmark of cancer [25,26]. Actually, cancer cells show impaired apoptotic signaling, which results in facilitating the development and metastasis of tumor [27,28]. In addition, the main goal of clinical oncology has been the development of therapies promoting the effective elimination of cancer cells by apoptosis for over several decades [29]. Therefore, the present study focused on the anticancer effect of coptisine on apoptosis in Hep3B cells and identified the action mechanism. Our findings showed that coptisine inhibited Hep3B cell viability and simultaneously increased apoptotic cell death.

DNA damage is a primary response with the biological goal of protecting a multicellular organism against damaged cells [30]. Faulty DNA damage-induced apoptosis resulted in tumorigenesis and in the resistance of cancer cells to defects for a variety of therapeutic agents [31]. DNA damage, including mitochondrial DNA strand breaks and degradation, provoke damage of lipids and proteins, which can cause apoptosis [32,33,34]. In the present study, we found that coptisine markedly promoted markers of DNA damage, such as an increase of comet tail length, TUNEL-positive cells, and the expression of p-γH2AX, indicating that DNA damage was accompanied by induction of apoptosis of Hep3B cells by coptisine.

It is well known that apoptosis is regulated by intrinsic and extrinsic signaling pathways that are triggered by multiple factors [29,35]. On the one hand, in the extrinsic pathway, apoptosis is induced by external signals, which directly activate caspase-8, and activated caspase-8 induces the activation of executioner caspases including caspase-3 and caspase-7 [35,36]. On the other hand, activation of the intrinsic pathway is associated with impaired mitochondrial function, regulated by internal signals, and linked to changes in the expression of Bcl-2 family proteins [35,37]. When the expression of proapoptotic members belonging to the Bcl-2 family proteins was increased as compared with the expression of anti-apoptotic members, mitochondrial permeability increased, and proapoptotic proteins, such as cytochrome *c*, were released into the cytoplasm. Cytochrome *c* released into the cytoplasm activated caspase-9, which, in turn, activated executioner caspases and led to the induction of apoptosis through the cleavage of various cellular substrates, such as PARP [38,39]. Our results show that coptisine induced the expression of DR4 and DR5 and activated caspases-8 and -9 corresponding to the initiator caspases of each apoptotic pathway. Coptisine also activated caspase-3 and induced the degradation of PARP, which was associated with the loss of MMP, upregulation of the Bax/Bcl-2 ratio, and cytosolic release of cytochrome *c*. Meanwhile, coptisine-induced apoptotic cell death was suppressed by Z-VAD-FMK, a pan caspase inhibitor. Therefore, based on those observations, we speculated that the proapoptotic effect of coptisine in Hep3B cells can be induced by simultaneously activating caspase-dependent extrinsic and intrinsic pathways.

Many previous studies have found that a number of anticancer drugs induce apoptosis through their oxidative properties, such as destroying cellular antioxidant systems or increasing ROS generation [40,41]. Although excessive ROS accumulation is closely related to mitochondrial dysfunction, it is involved in the activity of the intrinsic pathway, as well as the extrinsic pathway. In addition, the production of abnormal ROS is directly involved in causing oxidative damage of DNA [38,41]. Therefore, these observations suggest that an increase in intracellular ROS production in cells is one of the mechanisms for promoting the death of cancer cells. In the present study, we found that coptisine significantly increased the production of intracellular and mitochondrial ROS, whereas these accumulated ROS levels were markedly suppressed by NAC. Moreover, NAC clearly suppressed the effect of coptisine on apoptosis, such as the decreasing of cell viability, and upregelation of proapoptotic proteins and DNA damage. These findings demonstrate that coptisine-induced Hep3B cell apoptosis is a ROS-dependent phenomenon.

It is well know that the MAPKs signaling pathway regulates various cellular responses including apoptosis. Numerous studies have established that JNK and p38 MAPK signaling pathways are involved in cell death, whereas the ERK signaling pathway is associated with the survival of cells [42,43]. As reported, oxidative stress activates JNK and inactivates the anti-apoptotic genes, while it activates the proapoptotic genes of Bcl-2 family proteins [44]. ROS also activates JNK through inactivating JNK-inactivation phosphatase, followed by apoptosis [41]. In particular, Dhanasekaran et al. [45] reported that JNK stimulated the expression of proapoptotic proteins such as Bax and Bim including Bcl-2 homology (BH) 3 domain, suppressing anti-apoptotic activity by the phosphorylation of Bcl-2 at ser70 residues. In our results, coptisine increased the phosphorylation of JNK, but not ERK and p38 MAPK. Moreover, we found that ROS could act upstream of JNK in coptisine-stimulated Hep3B cells, and JNK inhibitor SP600125 reversed both upregulation of the expression on proapoptosis-related factors, and downregulation of the expression on anti-apoptosis-related factors, following by coptisine.

**Figure 8:**
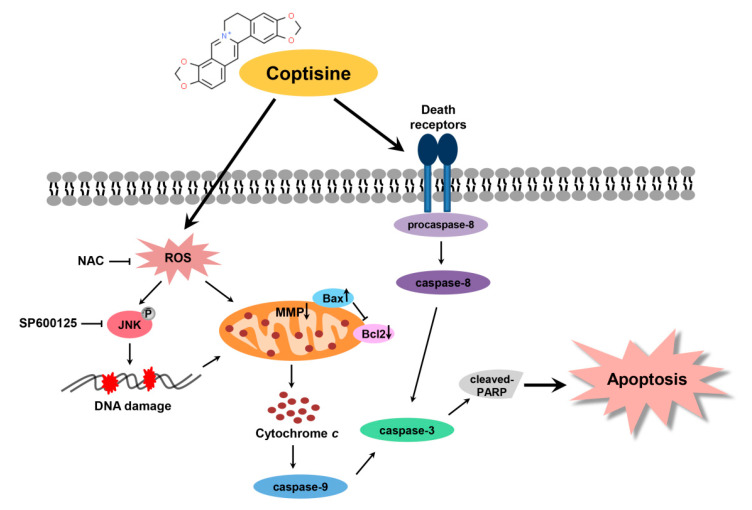
Schematic overview of apoptosis induced by coptisine via the induction of ROS-mediated JNK pathway in human HCC Hep3B cells.

In summary, the present study has demonstrated that coptisine exerts antitumor effects in Hep3B cells through induction of extrinsic and intrinsic apoptosis pathways. Coptisine induces ROS-dependent apoptosis by targeting JNK signaling pathway, DNA damage, and mitochondrial dysfunction (Figure 8). Taken together, the ability of coptisine provides information for understand the mechanism relevant to ROS in HCC cells death.

## 4. Materials and Methods

### 4.1. Cell Culture and Coptisine Treatment

The Hep3B cells were purchased from the American Type Culture Collection (Manassas, VA, USA). Cells were cultured in Dulbecco’s modified Eagle’s medium supplemented with 10% fetal bovine serum, 2 mM l-glutamine, and 1% antibiotics (100 U/mL penicillin and 100 µg/mL streptomycin; WELGENE Inc., Daegu, Republic of Korea) at 37 °C in a humidified 5% CO_2_ atmosphere. Coptisine (catalog No. SMB00314) was purchased from Sigma-Aldrich Chemical Co. (St. Louis, MO, USA), dissolved in dimethyl sulfoxide (pH 7.4, DMSO; AMRESCO, Dallas, TX, USA). Prior to use, the stock solution of coptisine was diluted to the required concentrations in culture medium.

### 4.2. Cell Viability

Cell viability was measured using an MTT assay, as previously described [46]. Hep3B cells were seeded on 96-well plate at a density of 5 × 10^3^ cells per well. After 24 h of incubation, the cells were treated with different concentrations of coptisine, for 24 h, in the presence or absence of NAC (Sigma-Aldrich Chemical Co.). Subsequently, the cells were incubated with 0.5 mg/mL MTT (AMRESCO) solution, at 37 °C. After incubation for 2 h, the medium was removed, and then 200 µL of DMSO was added to the well to dissolve the formazan. The cell viability was determined by measuring the optical density at 540 nm with a microplate reader (VERSA Max, Molecular Device Co., Sunnyvale, CA, USA).

### 4.3. Measurement of Apoptotic Cell Death by Flow Cytometric Analysis

The quantification of apoptosis was determined by flow cytometry using an annexin V-FITC/PI double staining kit (BD Pharmingen, San Diego, CA, USA), according to the manufacturer’s instruction. Briefly, cells were treated with the desired concentrations of coptisine, for 24 h, in the presence or absence of Z-VAD-FMK (Sigma-Aldrich Chemical Co.). The collected cells were re-suspended in binding buffer, and then stained with PI solution and FITC-conjugated annexin V, for 15 min, in dark, as previously described [47]. The fluorescence intensities were detected by flow cytometry (BD Biosciences, San Jose, CA, USA) at the Core-Facility Center for Tissue Regeneration, Dong-eui University (Busan, Repbulic of Korea).

### 4.4. Comet Assay for DNA Damage

The comet assay was assessed to detect the DNA damage in individual cells, as previously describe [48]. In brief, the collected cells were mixed with 0.75% low-melting agarose (LMA). The 70 µL mixture was transferred onto a CometSlide™ pre-coated with a layer of 0.75% normal-melting agarose, and then slides were placed in the dark, at 4 °C, for 40 min. The slides were immersed in lysis solution (2.5 M NaCl, 100 mM ethylenediaminetetraacetic acid (EDTA), 10 mM Tris, 1% Triton X-100, and 10% DMSO (pH 10)) in the dark, at 4 °C, for 60 min, and in alkaline unwinding solution, at room temperature (RT), for 20 min. The slides were placed in electrophoresis slide tray containing electrophoresis solution (300 mM NaOH, 10 mM Na-EDTA, pH 10), and electrophoresis was performed in the same solution, at 4 °C, for 30 min to draw the negatively charged DNA towards the anode. Next, the slides were gently immersed twice with a neutralization buffer (0.4 M Tris-HCl, pH 7.5) for 5 min, then in 70% ethanol for 5 min. After drying, cells were stained with SYBR green fluorescent dye, which binds to double-stranded DNA, and upon excitation emits light for 15 min in the dark, and then were observed by fluorescence microscopy (Carl Zeiss, Oberkochen, Germany). To prevent DNA damage, all of the steps were performed under yellow light. Tail length of the cells was analyzed by CometScore version 2.0 (TriTek, Los altos, CA, USA) software.

### 4.5. TUNEL Assay

The extent of the DNA breaks in apoptotic cells was evaluated using the TUNEL staining kit (Promega, Madison, WI, USA) [49]. In brief, cells were seeded onto glass coverslips in 4-well and treated with coptisine for 24 h. The cells were fixed with 4% methanol-free formaldehyde solution for 25 min, and then subjected to TUNEL assay, according to the manufacturer’s instructions. The cells were stained with PI for 10 min and the images were visualized using fluorescence microscopy.

### 4.6. Immunofluorescence for DNA Damage Detection

Cells were seeded in 4-well plates and treated as indicated in each experiment. After 24 h, cells were fixed with methanol at −20 °C for 10 min, and then washed three times using phosphate-buffered saline (PBS). The cells were permeabilized using 0.1% Triton X-100 (Sigma-Aldrich Chemical Co.) and blocked with 5% bovine serum albumin (BSA, Sigma-Aldrich Chemical Co.) in PBS, for 1 h. The cells were incubated with anti-p-γH2AX (Cell Signaling Technology, Beverly, MA, USA) antibody in 5% BSA overnight at 4 °C, and then washed with Tris-buffered saline and Tween 20 (TBST). Following washing for 1 h with anti-rabbit Alexa-conjugated secondary antibody (Abcam, Cambridge, UK) and staining with DAPI, they were observed by fluorescence microscopy.

### 4.7. Western Blot Analysis

The cells were collected, washed with PBS, and then lysed with lysis buffer, at 4 °C, for 30 min, to extract whole cell proteins [50]. The cytosolic and mitochondrial proteins were extracted using a mitochondria isolation kit (Thermo Fisher Scientific, Waltham, MA, USA). The protein samples were separated by SDS-polyacrylamide gel electrophoresis, and then transferred to PVDF membranes (Millipore, Bedford, MA, USA). Subsequently, the membranes were blocked with 5% BSA, for 1 h, in a dissolved mixture of TBST. Next, membranes were probed with the following primary antibodies overnight at 4 °C: anti-JNK, anti-p-JNK, anti-p-ERK, anti-p-p38 MAPK, anti-p-γH2AX (1:1000, all from Cell Signaling Technology, Massachusetts, Danvers, USA), anti-casepase-3, anti-caspase-8, anti-casepase-9, anti-PARP, anti-Bax, anti-Bcl-2, anti-Bid, anti-DR4, anti-DR5, anti-Fas, anti-cytochrome c, anti-p38, anti-ERK, anti-p-c-Jun, anti-VDAC, and anti-actin (1:1000, all from Santa Cruz Biotechnology, Santa Cruz, CA, USA). The membranes were incubated with anti-rabbit or anti-mouse peroxidase conjugated secondary antibodies (Santa Cruz Biotechnology, Inc.), for 2 h, at RT, and then washed three times with TBST. The membranes were developed by an enhanced chemiluminescent reagent (Amersham Biosciences, Westborough, MA, USA), and then photographed under a Fusion FX Image system (Vilber Lourmat, Torcy, France).

### 4.8. Caspase Activity

Caspase-3, -8, and -9 activities were determined using colorimetric assay kits (R&D Systems, Minneapolis, MN, USA), according to the manufacturer’s instruction. The cells were treated with coptisine for 24 h, and then cells were collected and lysed in the lysis buffer. After centrifugation, the supernatants were collected and incubated with the supplied reaction buffer, which contained the caspases substrates and dithiothreitol (Asp–Glu–Val–Asp (DEAD) for caspase-3, Ile–Glu–Thr–Asp (IETD) for caspase-8, and Leu–Glu–His–Asp (LEHD) for caspase-9), at 37 °C, for 2 h in the dark. The activities of caspases were determined by a microplate reader using wavelength of 405 nm.

### 4.9. Determination of MMP (Δψm)

The MMP was measured by JC-1 (Sigma-Aldrich Chemical Co.), which is a mitochondria-specific fluorescent dye. The cells were treated with the indicated concentrations of coptisine. After 24 h, the cells were subjected to 10 μM JC-1 dye, for 20 min, at 37 °C. After washing to remove unbound dye, the green (JC-1 monomers) and red (JC-1 aggregates) fluorescence was immediately acquired using flow cytometry.

### 4.10. Measurement of ROS Production

The production of intracellular and mitochondrial ROS was evaluated using DCF-DA (Sigma-Aldrich Chemical Co.) and MitoSOX™ red dye (Thermo Fisher Scientific), respectively. The cells were treated with coptisine for 30 min, and then incubated with 10 μM DCF-DA or 1 μM MitoSOX™ red, for 20 min, at 37 °C, without light. The fluorescence intensity was detected by flow cytometry. For image analysis of the ROS production, the cells were collected using a Thermo Shandon Cytospin 3 cytocentrifuge (Marshall Scientific, Hampton, NH, USA), and stained with DAPI, which is a nuclear-specific fluorescence dye. Subsequently, the cells were mounted using a mounting solution (Sigma-Aldrich Chemical Co.), and the images were visualized using an EVOS Cell Imaging System (Invitrogen, Carlsbad, CA, USA).

### 4.11. Statistical Analysis

All results were expressed as the mean ± SD. Statistical analysis was by a one-way analysis of variance (ANOVA) and Tukey’s post hoc test using GraphPad Prism software (version 5.03; GraphPad Software, Inc., La Jolla, CA, USA). The p-value was less than 0.05, and the differences between groups were considered statistically significant.

## 5. Conclusions

Overall, our results suggest that coptisine inhibited cell viability and simultaneously increased apoptosis that concerned both extrinsic and intrinsic pathways in Hep3B cells. In addition, coptisine induced DNA damage and mitochondrial dysfunction by the ROS-mediated JNK signaling pathway. Consequently, coptisine exerts an anticancer effect in Hep3B cells via the regulation of the ROS-induced JNK pathway, and this result provides further information for the treatment of HCC.
